# Enhanced antitumor efficacy through sustained release of 5-fluorouracil from poly(trimethylene carbonate) membranes

**DOI:** 10.3389/fphar.2024.1536764

**Published:** 2025-01-20

**Authors:** Zongheng Wang, Zhipeng Hou, Shuguang Zhang, Shuangyi Ren, Liqun Yang, Qianshi Zhang

**Affiliations:** ^1^ Department of Gastrointestinal Surgery, The Second Hospital of Dalian Medical University, Dalian, China; ^2^ Research Center for Biomedical Materials, Shenyang Key Laboratory of Biomedical Polymers, Engineering Research Center of Ministry of Education for Minimally Invasive Gastrointestinal Endoscopic Techniques, Shengjing Hospital of China Medical University, Shenyang, China; ^3^ Department of Thoracic Surgery, The First Affiliated Hospital of China Medical University, Shenyang, China

**Keywords:** poly(trimethylene carbonate), sustained drug release system, 5-fluorouracil, colorectal cancer, antitumor efficacy

## Abstract

**Introduction:**

Cancer remains one of the deadliest diseases worldwide. 5-Fluorouracil (5-FU) is one of the most commonly used chemotherapeutic agents in clinical practice today, but its effectiveness is often hampered by rapid drug metabolism and systemic toxicity. To address these limitations, the development of novel drug delivery systems for sustained drug release is essential to improve therapeutic outcomes.

**Methods:**

This study aimed to develop a poly(trimethylene carbonate) (PTMC) membrane capable of sustained drug release of 5-FU to enhance its antitumor activity. The membranes were prepared using the solvent-casting method, and their comprehensive properties were characterized.

**Results and Discussion:**

We evaluated the *in vitro* sustained release capability, as well as the biocompatibility and antitumor activity at both cellular and animal levels. The results demonstrated that the 5-FU-loaded PTMC membranes exhibited effective sustained release capabilities, superior biocompatibility, and enhanced antitumor effects compared to the 5-FU injections. These findings suggested that the 5-FU-loaded PTMC membranes hold great potential for application in cancer patients, offering benefits in chemotherapy treatment.

## Graphical Abstract

**Figure:**
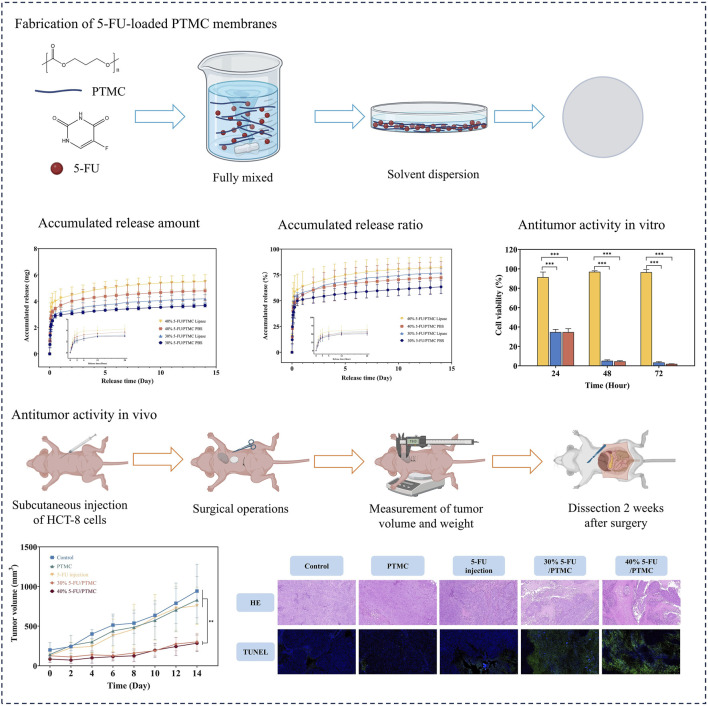

## 1 Introduction

As one of the most common malignant tumors of the digestive system, colorectal cancer has ranked third in incidence and second in mortality worldwide, making it a significant public health issue globally (Qiu et al., 2021; Sung et al., 2021). Currently, chemotherapy is one of the main approaches to prevent postoperative recurrence and extend cancer patients’ survival (Kuipers et al., 2015; Dekker et al., 2019). Among the agents, 5-Fluorouracil (5-FU) is a well-established medication commonly utilized in the treatment of colorectal cancer (Yang et al., 2024; Wang, 2020). It can inhibit DNA synthesis and lead to apoptosis of cancer cells (Longley et al., 2003; Yuan et al., 2021). However, treatment with 5-FU in chemotherapy faces certain challenges, such as a short half-life, significant cytotoxicity, and limited bioavailability (Entezar-Almahdi et al., 2020). Therefore, 5-FU is typically administered through multiple injections, which can exacerbate adverse reactions in patients (Vodenkova et al., 2020; Ozer et al., 2021). Studies have shown that continuous infusion of 5-FU offers higher efficacy and fewer side effects compared to high-dose injections (Li et al., 2018). Therefore, to address the limitations of 5-FU and improve its therapeutic efficacy, researchers have started exploring materials suitable for sustained drug release (Li et al., 2013; Leelakanok et al., 2018; Ren et al., 2012; Sun et al., 2013; Yusefi et al., 2021).

Poly (trimethylene carbonate) (PTMC) is an amorphous aliphatic polycarbonate known for its notable biodegradability and biocompatibility (Yu et al., 2021; Fukushima, 2016; Chen et al., 2014). It undergoes surface erosion degradation *in vivo* under the action of lipase, avoiding the rapid drug release triggered by the breakdown of the material (Zhang et al., 2006; Amsden, 2021; Kamaly et al., 2016). This characteristic overcomes the limitations of aliphatic polyesters such as polycaprolactone (PCL), polycaprolactone (PLA), polyglycolic acid (PGA), and their copolymers, making it more favorable for long-term sustained drug release. More importantly, PTMC does not generate acidic byproducts during its degradation process, preventing local pH reduction and subsequent inflammation (Yang et al., 2014; Hou et al., 2017; Hou et al., 2021). This characteristic ensures a stable and biocompatible environment for the drug release system. Consequently, PTMC and its applications have garnered widespread attention and research, highlighting its potential in advanced biomedical applications, particularly in developing efficient and reliable drug delivery systems.

PTMC had been successfully utilized as a coating for cardiovascular stents to enable stable drug release and enhance corrosion resistance (Tang et al., 2022). Additionally, it had been fashioned into membranes encapsulating antibiotics, serving as local antibiotic delivery systems (Kluin et al., 2009). It had also been developed into nanoparticles (Zhang and Zhuo, 2005), microspheres (Qiao et al., 2022; Dinarvand et al., 2006), and hydrogels (Wang et al., 2020) for drug delivery system. However, there are currently no studies on the use of PTMC as a sustained release carrier for 5-FU to enhance its antitumor activity.

Therefore, we employed the solvent-casting method to prepare 5-FU-loaded PTMC membranes. The sustained release performance of these membranes was tested through *in vitro* drug release experiments. Subsequently, we validated their biocompatibility and antitumor activity at both cellular and animal levels. The goal was to develop a novel delivery system for the long-term sustained release of anticancer drugs, aiming for improved anticancer efficacy.

## 2 Materials and methods

### 2.1 Chemicals and materials

5-FU was purchased from Adamas-beta (Shanghai, China) and used as received (CAS 51-21-8). TMC was sourced from Daigang Biomaterial Co., Ltd., recrystallized twice using acetic ether, and dried for 24 h at 37°C under reduced pressure prior to polymerization. Stannous octoate (Sn(Oct)_2_) (95%) was obtained from Sigma-Aldrich and prepared as a 0.1 M solution in toluene. Lipase solution (from Thermomyces lanuginosus, ≥100,000 U/g) was received from Sigma-Aldrich. Dichloride and methanol were obtained from Tianjin Damao Reagent Co. RPMI 1640 medium and Fetal bovine serum were from Gibco. Nude mice were purchased from Beijing Vital River Laboratory Animal Technology Co., Ltd.

### 2.2 Preparation of 5-FU-loaded PTMC membranes

The PTMC was fabricated through ring-opening polymerization, following the method described earlier (Yang et al., 2015; Hou et al., 2022). In brief, 30 g of TMC monomer and 146.92 μL of Sn(Oct)_2_ (0.1 M) were placed in a glass ampoule, vacuum sealed, and immersed in an oil bath at 130°C. After 24 h, the glass ampoule was allowed to cool to room temperature, thereby halting the polymerization reaction. The synthesized crude PTMC polymer was dissolved in dichloromethane and then precipitated in four times the volume of cold methanol for purification. The polymer was then vacuum-dried for later use.

Dissolve the preset equivalents of PTMC and 5-FU in appropriate amounts of dichloromethane and methanol solutions, respectively. Use a magnetic stirrer to thoroughly mix the solution, followed by ultrasonic treatment to evenly disperse the drug. Then, place the mixture into a flat-bottomed culture dish and evaporate it in a fume hood until the membrane is dry and its weight is constant. We made two different drug-loading concentrations of membranes, the 30% 5-FU-loading membranes and the 40% 5-FU-loading membranes, respectively.

### 2.3 Characterization of 5-FU-loaded PTMC membranes

The chemical structure of the membranes was analyzed using Fourier transform infrared (FT-IR) spectra and ^1^H nuclear magnetic resonance (^1^H NMR) spectra. The FT-IR analysis was performed using a Nicolet 6700 spectrometer (Thermo Fisher, United States), with a scanning wavenumber range of 4,000-400 cm^−1^. The ^1^H NMR spectra was recorded with a JNM-ECZ600R (JEOL, Japan) using dimethyl sulfoxide deuterated solution with tetramethyl silane as an internal reference. The SEM morphology of the 5-FU-loaded PTMC membranes were examined using a Sigma 300 (Zeiss, Germany). It was conducted at an accelerating voltage of 20 kV. Prior to testing, all specimens were coated with gold using an ion sputtering device to improve conductivity. The thermal properties of the samples were analyzed using a DSC 200 F3 (Netzsch, Germany) and a TGA 209 F3 (Netzsch, Germany). The DSC was equipped with a liquid nitrogen cooling system, and the temperature was scanned from −50°C to 280°C at a heating rate of 10°C/min under a nitrogen atmosphere. The TGA analysis was performed from room temperature to 600°C, with a heating rate of 10°C/min.

### 2.4 Loading efficiency and drug release for 5-FU-loaded PTMC membranes

The loading efficiency of the 5-FU-loaded PTMC membranes was measured using extraction. The 5-FU-loaded PTMC membranes were dissolved in dichloromethane, followed by the addition of ultrapure water for extraction. The concentration of 5-FU was measured using an ultra-high performance liquid chromatography (UPLC) system, Nexera LC-30A (Shimadzu, Japan). The mobile phase consisted of 95% deionized water and 5% methanol, with a flow rate of 0.1 mL/min. The detection wavelength was set at 265 nm, and the data acquisition time was 7 min. Each experiment was repeated three times, and the loading efficiency was calculated according to the following formulation.

$$Loading\;efficiency=\frac{Actual\;5-FU\;loaded\;membrane}{5-FU\;added\;membrane}\times 100\%$$

In the *in vitro* drug release study, 10 mL of PBS solution and 0.1% lipase solution were used as the release media. 5-FU-loaded PTMC membranes with a diameter of 5 mm were placed in them. At predetermined time points, 1 mL of the solution was withdrawn for UPLC analysis of drug concentration, and the fresh solution was added to maintain a constant volume. The PBS and lipase solutions were refreshed every 3 days.

### 2.5 Cell culture and proliferation

The human colorectal cancer cell line HCT-8 was obtained from Xiang Ya School of Medicine, Central South University. The cells were cultured in 1,640 medium (Gibco, Invitrogen) supplemented with 10% fetal bovine serum (Gibco, Invitrogen) and 1% penicillin-streptomycin (Gibco, Invitrogen) at 37°C in a humidified incubator with 5% CO_2_.

### 2.6 Determination of PTMC biocompatibility *in vitro*

The cytotoxicity of PTMC was assessed using the CCK8 assay. PTMC membranes discs with a diameter of 2 mm were sterilized with ultraviolet light and placed in 24-well plates. Each well was then filled with 2 mL of complete culture medium and incubated for 48 h. Subsequently, the culture medium was collected for further testing. HCT-8 cells were plated into 96-well plates at a density of 1 × 10^5^ cells/mL. After cell adhesion, 100 μL of the aforementioned culture medium was added to each well. The CCK8 assay was performed after 24、48、72 h to calculate cell viability.

### 2.7 Antitumor activity of 5-FU-loaded PTMC membranes *in vitro*

The *in vitro* antitumor activity of the 5-FU-loaded PTMC membranes was also evaluated using the CCK8 assay. Two concentrations of 5-FU loaded PTMC membranes with a diameter of 2 mm were sterilized with ultraviolet light and placed in 24-well plates. Each well was then filled with 2 mL of complete culture medium and incubated for 48 h. The culture medium was subsequently collected for further testing. The subsequent procedures were carried out as described in Section 2.6.

### 2.8 Animals preparation

Male BALB/C nude mice were purchased from Beijing Vital River Laboratory Animal Technology Co., Ltd. The experiments were conducted at the SPF Animal Experiment Center of Dalian Medical University. Before the experiments, the mice were allowed to acclimate for 1 week. The mice were kept on a 12-h light/dark cycle and had *ad libitum* access to food and water.

### 2.9 Antitumor activity and biocompatibility *in vivo*

An animal tumor model was established using human colorectal cancer HCT-8 cells. Each BALB/C nude mouse was administered a subcutaneous injection of 5 × 10^6^ HCT-8 cells into the axillary region. When the tumor volume reached 100–200 mm^3^, we divided the mice into five groups. Each group consisted three mice: a control group, a 5-FU injection group, a PTMC membranes group, a 30% 5-FU-loaded PTMC membranes group (140 mg/kg 5-FU), and a 40% 5-FU-loaded PTMC membranes group (220 mg/kg 5-FU).

The control group underwent a sham surgery, and the intraperitoneal injection group received 5-FU in saline every other day for a total of seven times, with a cumulative dose equivalent to the 30% 5-FU-loaded PTMC membranes group. For the mice in the PTMC and 5-FU loaded membrane groups, anesthesia was induced using tribromoethanol. The sterilized membranes were then implanted subcutaneously near the tumor. Finally, the incisions were closed using absorbable sutures. Following the surgery, the body weight and tumor volume of the mice were recorded every 2 days. The experiment concluded after 2 weeks. Tumor volume was calculated using the formula: $V=\left(\text{length}\times {\text{width}}^{2}\right)/2$ .

### 2.10 HE and TUNEL staining

At the conclusion of the animal experiments, the nude mice were euthanized using CO_2_ anesthesia. Tumor tissues were isolated for HE staining and TUNEL staining to assess tumor necrosis and apoptosis. Heart, liver, spleen, lung, and kidney tissues were collected from the nude mice and subjected to HE staining to check for any inflammation or damage. Additionally, the implanted membranes were retrieved from the animals for characterization, and the remaining drug content was measured.

### 2.11 Statistical analysis

All quantitative data are expressed as mean ± standard deviation, based on at least three replicates. Statistical analysis was conducted using SPSS software. Differences were deemed statistically significant at *p < 0.05, highly significant at **p < 0.01, and extremely significant at ***p < 0.001.

## 3 Results and discussion

### 3.1 The FT-IR and ^1^H NMR spectrums of pure PTMC and 5-FU-loaded PTMC membranes

**FIGURE 1:**
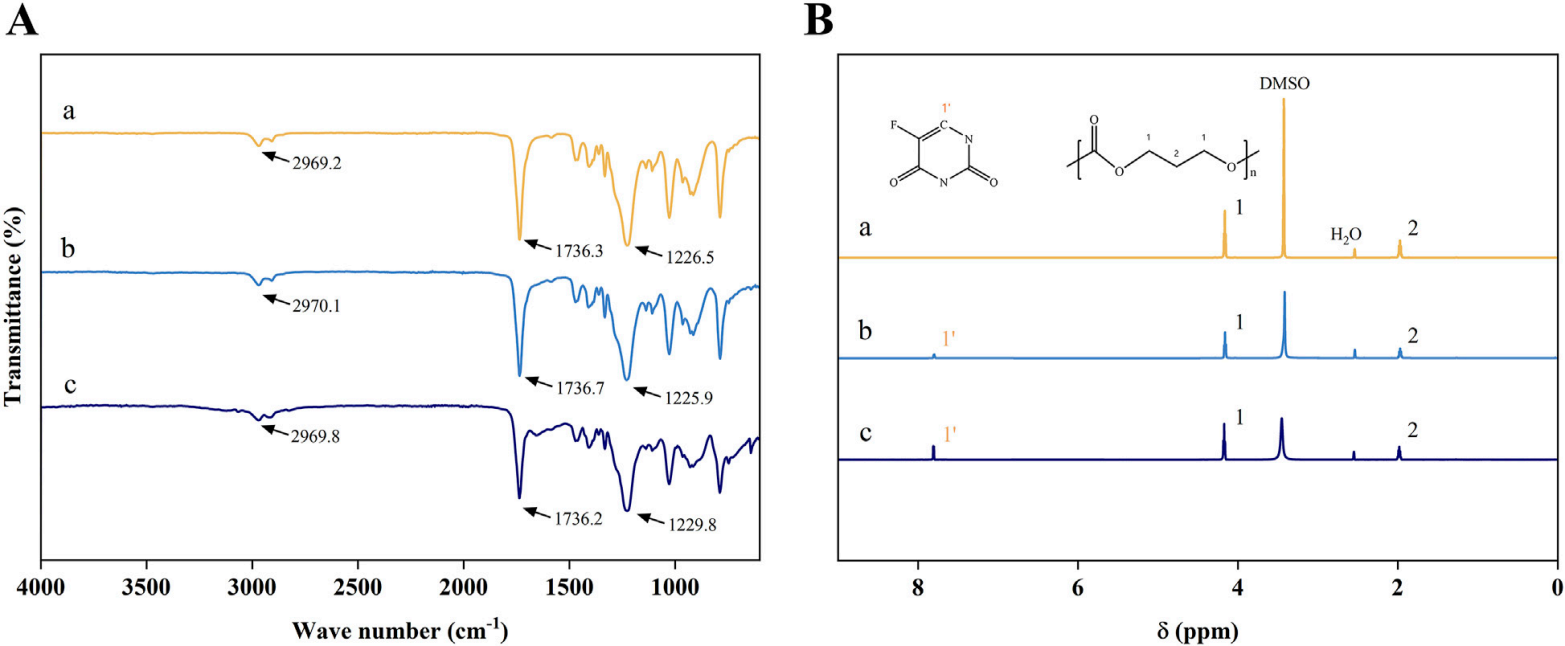
**(A)** The FT-IR spectrums; **(B)** The ^1^H NMR spectrums; (a), (b), and (c) represent the pure PTMC membranes, the 30% 5-FU-loaded PTMC membranes and the 40% 5-FU-loaded PTMC membranes.

Figures 1A, B presented the FT-IR and ^1^H NMR spectra of the pure PTMC membranes and the 5-FU-loaded PTMC membranes. The FT-IR results revealed the characteristic peaks of PTMC, including the C=O stretching vibration absorption peak at approximately 1736.3 cm⁻^1^, the C-O stretching vibration peak at around 1,226 cm⁻^1^, and the CH₂ stretching vibration peak at about 2,969 cm⁻^1^ (Han et al., 2010; Ren et al., 2021). Upon incorporation of different doses of 5-FU into the membranes, slight changes in the intensity of certain peaks were observed. However, the appearance of new peaks was minimal, indicating that the chemical structure of 5-FU remained intact within the PTMC membranes. This suggested that 5-FU and PTMC form a physical mixture with minimal chemical interactions.

In the ^1^H NMR spectrum, the proton peaks at positions 1 and 2 corresponded to the protons of -OCH_2-_ (chemical shift δ = 1.91 ppm) and -CH_2-_ (chemical shift δ = 4.09 ppm), confirming the correct structure of the compound (Zou et al., 2012). The chemical shifts of the proton peaks of deuterated DMSO and water were located at δ = 3.36 ppm and δ = 2.46 ppm, respectively. In the 5-FU-loaded PTMC membranes ^1^H NMR spectrums, the proton peak of 5-FU (chemical shift δ = 7.73 ppm) was observed, demonstrating that 5-FU has been effectively integrated into the PTMC membranes.

### 3.2 The SEM morphologies of pure PTMC and 5-FU-loaded PTMC membranes

**FIGURE 2:**
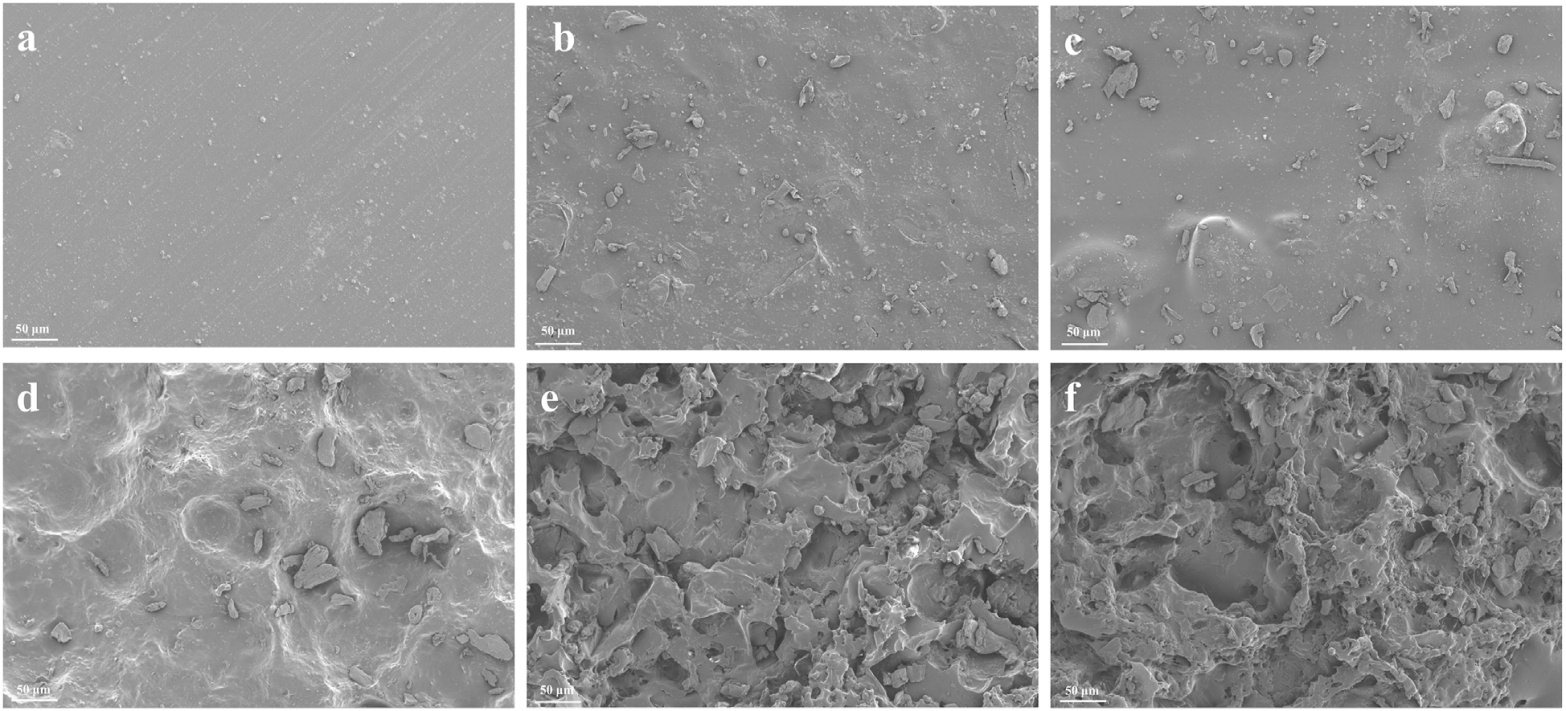
The SEM morphologies of pure PTMC and 5-FU loaded PTMC membranes; **(A–C)** represent the brand-new pure PTMC membranes, the 30% 5-FU-loaded PTMC membranes and the 40% 5-FU-loaded PTMC membranes; **(D–F)** represent the pure PTMC membranes, the 30% 5-FU-loaded PTMC membranes and the 40% 5-FU-loaded PTMC membranes after 2 weeks of implantation.

Figure 2 showed the SEM morphologies of each group. The surface of the brand-new pure PTMC membranes was smooth, while the 5-FU-loaded PTMC membranes exhibited rougher surfaces due to 5-FU incorporation. After 2 weeks of implantation in mice, all membranes exhibited noticeable porosity. This was consistent with the surface erosion degradation mechanism of PTMC (Wu et al., 2023; Yang et al., 2015). The 5-FU-loaded PTMC membranes exhibited more pronounced erosion and a greater number of cavities compared to the pure PTMC membranes after 2 weeks of implantation. This might be due to the drug release creating pores on the surface of the polymer matrix, which further increased the surface area of PTMC, thereby accelerating the degradation rate of the matrix.

### 3.3 The thermodynamic properties of pure PTMC and 5-FU loaded PTMC membranes

**FIGURE 3:**
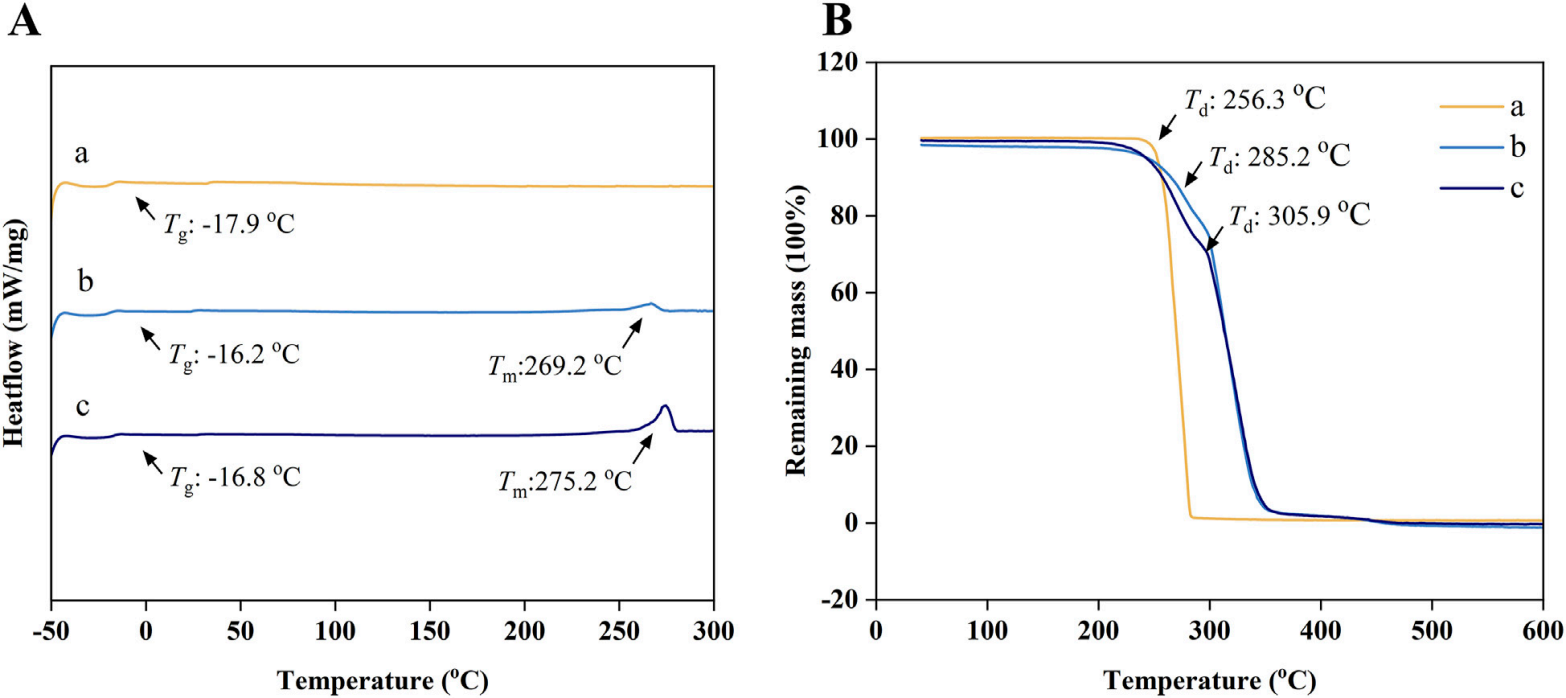
**(A)** The DSC curves; **(B)** The TGA curves; (a), (b) and (c) represent the pure PTMC membranes, the 30% 5-FU-loaded PTMC membranes and the 40% 5-FU-loaded PTMC membranes.

Figures 3A, B present the DSC and TGA results of pure PTMC membranes and 5-FU-loaded PTMC membranes. DSC analysis revealed that the glass transition temperature (Tg) of pure PTMC was measured at −17.9°C, indicating suitable elasticity and flexibility at human physiological temperature. After incorporating 5-FU into PTMC, a melting peak of the 5-FU could be observed around 270°C, confirming that the drug was successfully incorporated into the PTMC. However, the incorporation of 5-FU did not significantly alter the glass transition temperature, implying that the drug and polymer primarily form a physical blend without altering the internal structure of PTMC, and thus the PTMC membranes maintained their flexibility.

The TGA results indicated that the thermal decomposition temperature (*T*_d_) of pure PTMC is 256.3°C. Moreover, the decomposition temperature increased with the incorporation of 5-FU, suggesting enhanced thermal stability.

Many current drug delivery systems exhibit thermal instability, like liposomal nanoparticles that decompose and lose their functionality at elevated temperatures, which poses a significant challenge for efficient sterilization (Domanska et al., 2020). The thermal stability of PTMC and 5-FU enables the membranes to withstand high-temperature and high-pressure sterilization processes, thereby promoting their widespread clinical application.

### 3.4 The loading efficiency and release study for 5-FU-loaded PTMC membranes

The drug loading efficiency of the membranes was determined by extraction. The 30% 5-FU-loaded PTMC membranes and 40% 5-FU-loaded PTMC membranes achieved drug-loading efficiencies of 94.9% and 88.1%, respectively, indicating the strong capacity of PTMC for drug incorporation.

**FIGURE 4:**
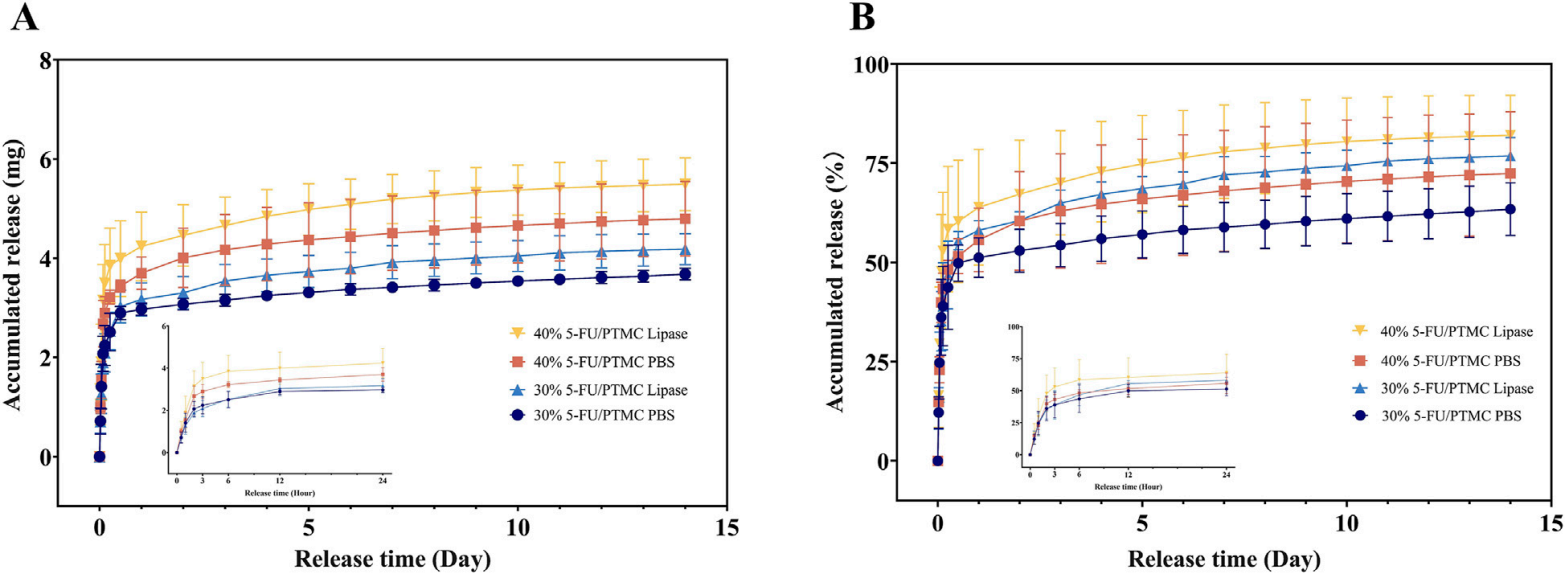
The release study for 5-FU-loaded PTMC membranes *in vitro*. **(A)** The accumulated release amount; **(B)** The accumulated release ratio.

Figure 4 showed the *in vitro* drug release results of the 5-FU-loaded PTMC membranes. After an initial burst release of 6 h, the 5-FU-loaded PTMC membranes were able to release 5-FU continuously over 2 weeks, demonstrating effective sustained release capability. In the presence of lipase, the release rate of 5-FU increased, and the total amount released was also higher. This was attributed to the accelerated degradation of PTMC in the presence of lipase, enhancing drug release (Zhang et al., 2005).

Additionally, we measured the residual 5-FU content in the membranes retrieved from the mice. The results showed that the 30% 5-FU-loaded PTMC membranes and 40% 5-FU-loaded PTMC membranes had residual drug amounts of 0.13 mg and 0.24 mg, respectively, indicating that the drug-loaded membranes achieved ideal release over the 2 weeks.

This indicates that the PTMC membranes only need to be implanted once to achieve a prolonged and stable release of 5-FU in the body. This feature effectively overcomes the limitation of the short half-life of 5-FU. In contrast, nanoparticle carriers, such as liposomes, are usually dependent on the external environment, such as pH, to regulate the release rate, and the release process is fast, making it difficult to achieve long-term release, thus requiring multiple injections, which increases the inconvenience and burden of treatment for patients in clinical applications.

### 3.5 Determination of PTMC biocompatibility and antitumor activity of 5-FU-loaded PTMC membranes *in vitro*

**FIGURE 5:**
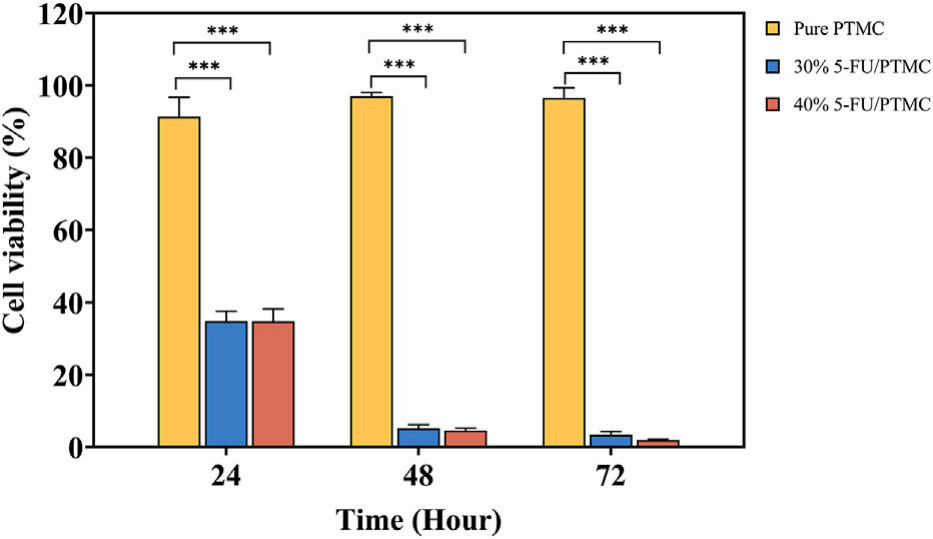
The CCK8 results of Pure PTMC and 5-FU-loaded PTMC membranes, ***p < 0.001.

The CCK8 assay was performed in triplicate with five replicates per experiment. The cell viability of cells cultured in pure PTMC extract relative to those in blank culture medium was calculated. As shown in Figure 5, the viability rates at 24, 48, and 72 h were 91.41%, 96.99%, and 96.54%, respectively, indicating PTMC membranes had minimal impact on cell proliferation.

For the 5-FU-loaded PTMC membranes, cells were cultured in a medium preconditioned with them. The viability rates for the 30% 5-FU-loaded PTMC group were 34.82%, 5.20%, and 3.41% at 24, 48, and 72 h, respectively. In the 5-FU-loaded PTMC group, the viability rates were 34.75%, 4.58%, and 1.95% at the same time points. The results of *in vitro* cell experiments demonstrated that PTMC membranes loaded with 5-FU effectively suppressed tumor cell proliferation.

### 3.6 Antitumor activity and biocompatibility *in vivo*

**FIGURE 6:**
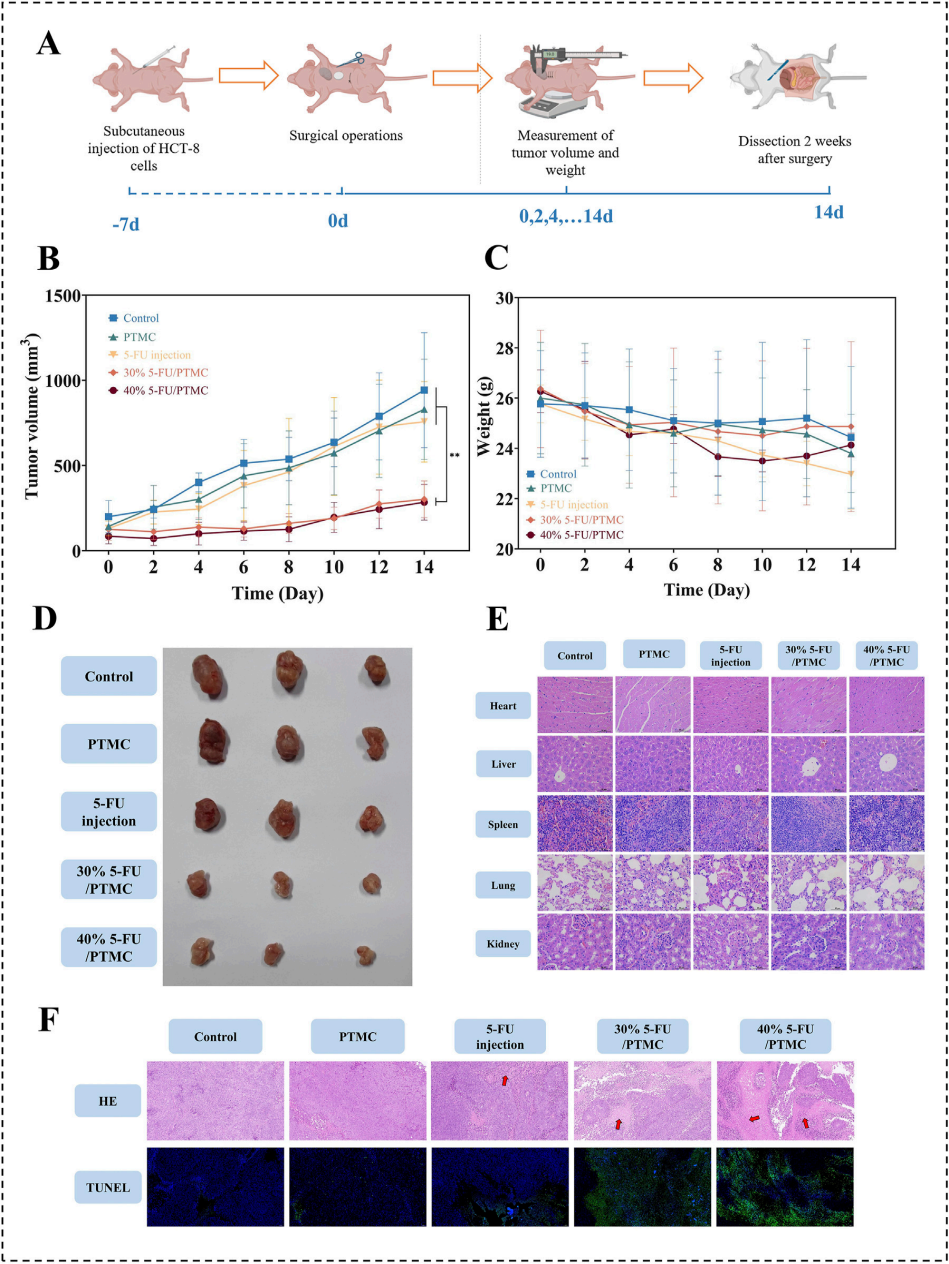
**(A)** Animal experiment process; **(B)** Changes in tumor volume over 2 weeks post-surgery, **p < 0.01; **(C)** Changes in body weight of mice over 2 weeks post-surgery; **(D)** Tumors excised from mice after 2 weeks of implantation; **(E)** HE staining of major organs; **(F)** HE and TUNEL staining of tumor tissues, the red arrowed regions indicated areas of tumor necrosis (n = 3).

Figure 6A showed the process of animal experimentation. Approximately 1 week after injecting the tumor cells, we performed the implantation surgery. Figure 6B showed the changes in tumor volume over the 2 weeks following surgery, and Figure 6D displayed the tumors excised from the mice after 2 weeks of implantation. It was evident that the tumor volume in the 5-FU-loaded PTMC membranes groups was substantially smaller compared to the 5-FU injection group, demonstrating the better antitumor activity of the 5-FU-loaded PTMC membranes *in vivo*. This was mainly because the 5-FU-loaded PTMC membranes could continuously release the drug, overcoming the poor therapeutic effect caused by its short half-life.

Additionally, tumor tissues from each group were subjected to HE and TUNEL staining to observe tumor necrosis and apoptosis, as shown in Figure 6F. In the HE-stained sections, the red arrowed regions indicated areas of tumor necrosis. It revealed significantly larger necrotic areas in the tumors of the 5-FU-loaded PTMC membranes group compared to the other groups. In the TUNEL-stained sections, the green color represented apoptotic tumor cells. TUNEL staining indicated more pronounced apoptosis in the 5-FU-loaded PTMC membranes group. This indicates that the 5-FU-loaded PTMC membranes can significantly promote tumor cell necrosis and apoptosis compared to the intraperitoneal injection group.

Figure 6C showed the changes in the body weight of the mice. The 5-FU-loaded PTMC membranes groups showed no significant weight loss compared to the control group. At the end of the experiment, HE staining was performed on the heart, liver, spleen, lungs, and kidneys of each group to assess inflammation and necrosis. As shown in Figure 6E, the HE staining results showed no significant inflammation or tissue necrosis in any group, confirming the biocompatibility of the 5-FU-loaded PTMC membranes *in vivo*.

## 4 Conclusion

To achieve sustained release of 5-FU *in vivo*, we successfully prepared 5-FU-loaded PTMC membranes using the solvent-casting method. The membranes exhibited suitable elasticity and flexibility at human physiological temperature, making them suitable for implantation in the body. The *in vitro* experimental demonstrated that they have favorable sustained release capability, consistently releasing the drug over 2 weeks. Both cell and animal experiments indicated their significant biocompatibility. Animal experiments showed that compared to the 5-FU injection group, the 5-FU-loaded PTMC membranes exhibited superior tumor growth inhibition and more effectively promoted tumor necrosis and apoptosis. Therefore, we propose that the 5-FU-loaded PTMC membranes can serve as a sustained drug release system that enhances the chemotherapy effectiveness of 5-FU, providing potential benefits for postoperative chemotherapy treatment in cancer patients.

## Data Availability

The original contributions presented in the study are included in the article/supplementary material, further inquiries can be directed to the corresponding authors.
